# Can Households Cope with Health Shocks in Vietnam?

**DOI:** 10.1002/hec.3196

**Published:** 2015-05-28

**Authors:** Sophie Mitra, Michael Palmer, Daniel Mont, Nora Groce

**Affiliations:** ^1^ Fordham UniversityNew YorkNYUSA; ^2^ University of MelbourneMelbourneVictoriaAustralia; ^3^ University College LondonLondonUK

**Keywords:** health shocks, disability, morbidity, coping mechanisms, expenditures, Vietnam

## Abstract

This paper investigates the economic impact of health shocks on working‐age adults in Vietnam during 2004–2008, using a fixed effects specification. Health shocks cover disability and morbidity and are measured by ‘days unable to carry out regular activity’, ‘days in bed due to illness/injury’, and ‘hospitalization’. Overall, Vietnamese households are able to smooth total non‐health expenditures in the short run in the face of a significant rise in out‐of‐pocket health expenditures. However, this is accomplished through vulnerability‐enhancing mechanisms, especially in rural areas, including increased loans and asset sales and decreased education expenditures. Female‐headed and rural households are found to be the least able to protect consumption. Results highlight the need to extend and deepen social protection and universal health coverage. © 2015 The Authors. *Health Economics* published by John Wiley & Sons Ltd.

## 1 Introduction

Idiosyncratic shocks in low‐income and middle‐income countries (LMICs), especially health shocks, are both common and burdensome (Heltberg and Lund, 2009; Krishna, 2010; Santos *et al*., 2011; Wagstaff and Lindelow, 2014). They may reduce income, because of lost hours of work, and increase health expenditures. In the absence of formal insurance mechanisms, the economic consequences of health shocks for the household can be dire. As many LMICs commit to achieving universal health coverage (Giedion *et al*., 2013), understanding the impact of health shocks is critical to inform the desirability and design of social health protection programs.

Much of the empirical work on health shocks, and on income shocks in general, has focused on assessing if households can insure total consumption when shocks occur (e.g., Gertler and Gruber, 2002). While early studies found that consumption was stable despite shocks (Townsend, 1994), later studies have shown that consumption drops can be significant (e.g., Gertler and Gruber, 2002), especially among certain subgroups such as people living in poverty (Morduch, 1995).

At a theoretical level, Chetty and Looney (2006) show that consumption is not an adequate indicator for the need for social insurance, insofar as households may maintain consumption because of risk aversion. They may do so using various strategies, including substituting labor within the household, drawing upon savings or informal family support networks, turning to higher‐risk strategies of taking out interest‐bearing loans, removing children from school, and selling productive assets (Sauerborn *et al*., 1996). The effect of these strategies on the welfare of households over time is currently not well understood. Another concern is that studies generally assess the average impact of health shocks at the population level without disaggregation (e.g., Gertler and Gruber, 2002). Different types of households are likely to have different access to coping mechanisms. For example, poor households may have less savings and lower levels of social capital; single‐parent or smaller‐size households may have less ability to substitute labor within the household.

Using three waves of the Vietnam Household Living Standards Survey (VHLSS) and three health shock measures, we address the following key questions in this study:

1. How do household income and health spending respond to health shocks?
2. Is household non‐health consumption – including food consumption – protected against health shocks?
3. What coping mechanisms are used in response to health shocks?

As often noted (e.g., Currie and Madrian, 1999; Strauss and Thomas, 2008), health is a concept everyone seems to understand and yet is complex, multifaceted, and difficult to define and measure. In this paper and more broadly in economics, the term ‘health shocks’ is often used to refer to negative health events, which are unexpected health events but can also include expected lasting permanent or recurring illness and disability. This paper uses several health shock measures including ‘days unable to carry out regular activity because of illness/injury’, ‘days in bed due to illness/injury’, and ‘hospitalization’.

In this paper, we find that health shocks lead to a large increase in out‐of‐pocket health expenditures. We also find that households are generally able to insure total consumption in the face of this increase in medical spending yet with considerable variation across population groups. Female‐headed households and, to a lesser extent, rural households are the least able to insure consumption. Across the population, we find the use of coping strategies to maintain current consumption, in particular increased loans and asset sales and decreased education expenditures. While helping to maintain current consumption levels, such strategies are likely to compromise the future welfare of households. Public and private transfers play a small to insignificant insurance role.

The paper is organized as follows. Section 2 reviews the relevant literature. Section 3 describes the data and the measures. Section 4 presents the empirical strategy. Section 5 includes results, and Section 6 has discussion of results and policy implications for Vietnam and, more broadly, other LMICs. Section 7 concludes.

## 2 Background

### 2.1 The literature on health shocks and household welfare

While the importance of health to economic and social development is widely recognized, the body of evidence on the effect of health on economic and welfare outcomes is limited, particularly for LMICs. This is due to the challenges that have been explained in detail elsewhere (Strauss and Thomas, 1998). First, there is the challenge of measuring health changes, as they can take many forms and vary in levels of intensity.

In economics, eight health measures have been commonly used (Currie and Madrian, 1999): (1) self‐reported health status (most often whether someone is in excellent, good, fair, or poor health); (2) whether there are health limitations on the ability to work; (3) whether there are other functional limitations such as problems with activities of daily living (ADL); (4) the presence of chronic and acute conditions; (5) the utilization of medical care; (6) clinical assessments of such things as mental health or alcoholism; (7) nutritional status (e.g., height, weight, or body mass index); and (8) expected or future mortality. This paper uses measures related to (2), (3), and (5) mentioned earlier, as follows: ‘days unable to carry out regular activity because of illness/injury’, ‘days in bed due to illness/injury’, and ‘hospitalization’.

There are very few representative surveys from LMICs that track different health facets and severity levels over time. It is also important to note that even when measurements are available, endogeneity may be a problem. This endogeneity can arise from measurement errors in health and economic's well‐being that may be correlated with characteristics that are often included in models such as age and education. Measurement errors may be systematic or random. Second, there is unobserved heterogeneity that affects both health and economic welfare. For instance, there may be unobserved factors that affect economic welfare and may be correlated with health (e.g., early childhood nutrition). Unobserved factors may be time‐invariant or time‐varying. Third, it is difficult to establish causality as there is likely two‐way causation between health and economic welfare. Feedback effects from economic welfare to health may be positive (e.g., through the purchase of health inputs, including better nutrition and healthcare) or negative (e.g., through the purchase of assets such as motorbikes that lead to more injuries). Thus, health shocks are not strictly exogenous.^1^

Different econometric specifications have been used to measure the impact of health on welfare. Earlier works use ordinary least squares (OLS) (Kochar, 1995). However, they do not address the challenges outlined earlier with respect to a measurement error bias, an omitted variable bias, and reverse causality. To address reverse causality, some studies use a lagged specification where a health shock in the previous period affects the welfare in the current period (e.g., Wagstaff, 2007a). While this may address reverse causality to some extent, it does not address an omitted variable bias or a measurement error. Other studies have used a first difference or fixed effects (FE) specification, which has the advantage of differencing out a systematic measurement error and time‐invariant unobserved heterogeneity (Gertler and Gruber, 2002). However, as pointed out by Strauss and Thomas (2008), there may be a random measurement error or unobserved heterogeneity that varies over time, which is not addressed by first differencing and may be of particular concern for health measures.^2^ Recently, Genoni (2012) used a first difference specification with instrumental variables (IVs) in an attempt to address a random measurement error in a study of mostly household‐level outcomes, focusing on a few specific physical limitations as health measures.

Despite these econometric challenges, the literature on the impact of health shocks on consumption and income has been growing. Its results are mixed overall. Several studies have found that health shocks negatively affect household consumption and income (Dercon and Krishnan, 2000; Gertler and Gruber, 2002; Lindelow and Wagstaff, 2005; Wagstaff, 2007a; Somi *et al*., 2009; Wagstaff and Lindelow, 2014),^3^ whereas others have found no statistically significant effect (Townsend, 1994; Genoni, 2012; Islam and Maitra, 2012). Effects have been found to vary across subgroups and health measures. Welfare effects of health shocks have been found to be more pronounced on the poor (Dercon and Krishnan, 2000), urban areas (Wagstaff, 2007a), and low‐educated households (Genoni, 2012). As for the differences in outcomes related to the health measures examined, Gertler and Gruber (2002), for instance, find that households are able to fully insure health shocks as measured by illness symptoms but are unable to insure those measured by limitations in the ability to perform ADL.^4^

Fewer studies have examined the coping mechanisms associated with health shocks in LMICs. In a qualitative study in Burkina Faso, Sauerborn *et al*. (1996) find that intra‐household labor substitution is the main coping strategy after the onset of an illness, and yet, this did not eliminate production losses in the majority of households. Other studies have also inferred labor substitution within the household. For example, in Vietnam, Wagstaff (2007a) finds higher effects of a death on earned income in urban areas, which are attributed to households in rural areas being better able to adjust labor supply. Unearned income (gifts, remittances, and pensions) is found to partially offset losses in earned income in Vietnam and does so more in urban areas (Wagstaff, 2007a).

To our knowledge, only three recently published studies using nationally representative data sets consider the mechanisms to cope with health shocks other than changes in household labor supply and unearned income in LMICs. First, Genoni (2012) studies the sale of ‘liquid’ assets (durables, financial wealth, and jewelry) and remittances from relatives in Indonesia. The author finds remittances cushion consumption, whereas asset sales are not responsive to health shocks. Second, still for Indonesia, Sparrow *et al*. (2013) find that borrowing and drawing on family network and savings/assets are the main coping strategies for the poor. Third, Islam and Maitra (2012) find that Bangladeshi households are able to insure consumption through the sale of livestock and access to microcredit.

With the exception of the work of Islam and Maitra (2012), which adopts an IV strategy of eligibility to microfinance, the potential endogeneity of coping mechanisms is not addressed in the studies of coping mechanisms in health. It is possible that unobservable factors (e.g., social capital) that influence whether a household has access to transfers, credit, or other coping mechanisms are also correlated with measures of household welfare, leading to biased estimates of the impact of coping mechanisms on outcomes.

In the case of Vietnam, the recent evidence on the economic consequences of health shocks is limited. Nguyen *et al*., (2012) study the coping strategies used by residents of Dai Dong, a rural commune of Hanoi, and found that households were more likely to undertake loans or reduce food consumption to pay for medical treatments, especially among those who were classified as poor. A working paper by Wainwright and Newman (2011) uses data from rural Vietnam to assess a variety of shocks, including a health shock defined as any family member suffering an illness, an injury, or death. Their results suggest that households deplete their stock of total liquid assets in the event of exposure to shocks, including health shocks. It is unclear, however, what type of health shocks is captured with a broad self‐report of whether ‘any family member suffered from an illness, an injury, or death.’ Bales (2013) starts to deal with the economic consequences of health shocks using nationally representative data, but uses health measures with limited variation in the data (bedridden for 14 or more days, death of working‐age adult) or with observations at only one point in time (functional limitations), which prevents the estimation of a FE model.

This paper uses nationally representative data and a FE specification to measure the consequences of health shocks on a wide range of household economic welfare outcomes in Vietnam. The paper contributes to the understanding of the economic consequences of health shocks in LMICs in three ways. First, we use a broader array of welfare outcomes and coping mechanisms compared with earlier papers. Second, results are presented for the general population and specific subgroups to assess vulnerability for the different types of households. Third, our study period coincides with a period of expansion of the social protection and health insurance system in Vietnam; hence, the findings have implications for on‐going reforms to the system.

### 2.2 Social protection in Vietnam

Transitioning from a centrally planned to a market‐based economy, Vietnam has achieved high economic growth and a remarkable reduction in the rate of poverty over the past two decades. In 2010, the country crept into the lower middle‐income country bracket with a per capita income of $1130. Rapid economic transformation and growth, however, have contributed to rising inequality in income and access to education, employment, and health service opportunities. Disparities have been recorded between rural and urban areas, the poor and the rich, ethnic minorities and the majority, men and women, people with and without disabilities, and regions (Pham and Reilly, 2007; Mont and Nguyen, 2011; Kang and Imai, 2012; Le and Booth, 2014).^5^ Furthermore, while large numbers of households have moved out of poverty in recent years, many have not moved far above the poverty line and remain vulnerable to shocks in the macroeconomic policy environment, weather, and health (Baulch and Vu, 2010).

To address these concerns, the government has over time built a system of social protection support for social beneficiary groups alongside a broader social health insurance system. Two key safety net programs include an unconditional cash transfer program and a non‐contributory health insurance program for the poor and social beneficiary groups (e.g., ethnic minorities, orphans, persons with disabilities, and the elderly living alone) (Decree 67, 2007). The remainder of the population is eligible for contributory health insurance through a compulsory or voluntary scheme.

Earlier work by Wagstaff (2007a) uses 1993–1998 data that absorbs the introduction of national compulsory and voluntary social health insurance schemes in 1992 and 1994, respectively. However, the health insurance system in Vietnam has undergone significant changes since 1998.^6^ In 2003 and more substantially in 2006, the non‐contributory social beneficiary health insurance scheme was extended under a new funding arrangement (known as the Health Care Fund for the Poor) to target households classified as poor, ethnic minorities in selected mountainous provinces, and households in especially socioeconomically disadvantaged communes. From 2005, children under the age of 6 years were added to the list of non‐contributing groups. Over the 4‐year period, 2002–2006, insurance coverage of the population increased from approximately 14% to 42% (Lieberman and Wagstaff, 2009). The list of reimbursable items under insurance has also grown steadily over time, and so has the monetary amount of public cash transfers.

While this study examines the impact of health shocks against a background of recent reforms to the social protection system, its aim is not to isolate the effects of particular policy reforms. Instead, it focuses on health shocks in the entire working‐age population. The working‐age population is not a target group for social protection purposes and yet is a group whose health status is intuitively most likely to affect the economic welfare of the household. In fact, large numbers of the informal work sector, including farmers and the self‐employed, remain currently uninsured in Vietnam (Somanathan *et al*., 2013). These near poor are also not eligible for public cash transfer supports unless they fall under a social beneficiary group category as defined in Decree 67 (2007).

## 3 Data and Measures

The data used in this study come from three waves of the VHLSS, a large‐scale socioeconomic survey conducted in Vietnam. The three waves cover the years 2004, 2006, and 2008: Only 50% of the households interviewed in 2004 are retained in the 2006 wave, and 50% of households in 2006 are retained in 2008. These three waves form a panel of 1552 households. The survey links individual, household, and community‐level data, bringing together an enormous amount of information that enables us to better isolate the impact of health changes on household welfare outcomes.

The VHLSS has several types of health shock measures. It measures morbidity through self‐reported symptoms such as nausea, fever, or coughing. We elect not to use morbidity measures because of concerns of systematic and random measurement errors. Respondents may interpret these symptoms differently, and their interpretation is influenced by, and in turn influences, healthcare use. As Strauss and Thomas (1998) note in the LMIC context, ‘it is not unusual for the poorest to appear to be the most healthy by this metric!’ (p. 791). The VHLSS also collects information on physical functioning through questions on difficulties in ADL, such as walking a specific distance or bending. ADLs are specific and less likely to suffer from a measurement error than morbidity (Dow *et al*., 1997). However, physical functioning data are available only in the 2006 wave of the VHLSS and thus cannot be included in this study, which adopts a panel design. We elect to use a measure of ‘days of regular activity lost because of illness/injury’ and a measure of ‘days in bed due to illness/injury’. These two measures may capture the person's level of functioning in the lived environment and may thus be the measures of disability as activity limitations and participation restrictions under the International Classification of Disability and Health (World Health Organization, 2001). They may also reflect ill health, in particular episodes of acute illness (e.g., malaria and flu).

Following Wagstaff (2007a, 2007b), we also include the hospitalization of a working‐age household member in the previous 12 months as an indicator of a health shock. It is of interest to compare the results of this earlier study, which uses data from the 1990s. Wagstaff (2007a) also uses the death of a working‐aged member. However, in this data set, the number of households experiencing the death of a working‐age member was very low (53 over the three data waves). Furthermore, as Grimm (2010) notes, mortality shocks are distinct from health shocks on their impact on household welfare, because of the compensating effects of a reduction in the number of consumption units.

We use health shock status only for working‐age members (15–60 years) as they are more likely to affect household welfare outcomes. It is important to note that these measures reflect time allocation decisions that can be influenced by the wage or other work‐related factors (e.g., working conditions) and therefore are endogenous with employment. This is especially problematic for the study of individual labor market outcomes. Therefore, this study will not cover individual‐level employment outcomes and instead will focus on household‐level welfare outcomes.

We analyze a comprehensive range of economic welfare outcomes as dependent variables: household expenditure (total, food, non‐food, education, and medical^7^) and income (earned and unearned) outcomes. Unearned income is divided into private transfers, public transfers, asset sales, borrowings, and savings withdrawal to provide an analysis of coping mechanisms. Private transfers comprise domestic and international remittances and donations from charities. Public transfers include pensions, social welfare, and retirement allowances.

## 4 Empirical Strategy

As a theoretical framework underlying the analysis in this paper, we use an intertemporal consumption model with income uncertainty (e.g., Deaton, 1992). It is assumed that households are risk‐averse and maximize intertemporal expected utility defined in terms of consumption. In such a framework and if risk sharing or consumption smoothing is possible, idiosyncratic shocks on income, such as an illness or a disability, leading to hospitalization or an inability to work are smoothed out. However, if risk sharing and consumption smoothing are imperfect, transitory shocks on income will alter consumption. For instance, the injury of a household member could, via a possible effect on high medical expenditure, reduce the resources available for consumption. Households could cope with such a shock by selling assets, adjusting or withdrawing from savings, or undertaking credit. This paper estimates the reduced form relationships between health shocks, on the one hand, and household consumption and coping mechanisms, on the other. In this estimation, health shocks are not considered as exogenous.

As in the dynamic health production function of Grossman (1972), current health status is a function of current and past health inputs, labor supply, and the environment. There are at least three sources of heterogeneity that may be correlated with health inputs and economic welfare outcomes: time‐invariant, time‐varying unobserved heterogeneity, and a measurement error in health and welfare.

While we mainly present the results of a FE model, we start with a pooled OLS specification as follows:

(1) $$\ln Y_{h,t}=\beta_{0}+\beta_{1}\;H_{h,t}+\beta_{2}X_{h,t}+\beta_{3}\;Z_{h}+\propto_{t}+\propto_{c}+\;\delta_{c,t}+u_{h,t}+u_{h}\text{,}$$

where *h* indexes households, *c* communities, and *t* time periods; *Y* denotes the economic welfare outcome; *H* is the health shock variable; and *β*_1_ is its coefficient. Following the conceptual framework outlined by Strauss and Thomas (1998), we include a set of controls at the household‐level X, which are relevant and the time‐varying determinants of welfare: characteristics of the household (household size, shares of members under age 16 and over 60 years, share of male household members, living in a remote commune,^8^ month of interview, and whether the household experienced a change in the household head) and characteristics of the household head (age, marital, and education status).

We also include a vector of controls ***Z***, which are relevant and the time‐invariant determinants of welfare: urban area, provincial region, and the gender and ethnicity of a household head. We include time and community FEs^9^ and account for the changes in community‐level labor markets by community‐time interaction terms. Without community time interaction terms, the regression may yield biased estimates because of the possible correlation between the omitted or unobserved time‐varying community characteristics and the error term. It also allows us to control for any aggregate or covariate risks faced by all individuals in the community, including price changes that are community‐specific over time and community‐level shocks. The terms *u*_*h*,*t*_ and *u*_*h*_ are household‐specific error terms that account for time‐varying and time‐invariant unobservables for the household, respectively.

Given the availability of panel data, we considered using a random or FE model to attempt to address endogeneity. We conducted Haussman tests that strongly rejected the null hypothesis that random effects provide consistent estimates,^10^ and thus adopted the household FE specification as our main specification as follows:

(2) $$\ln Y_{ht}=\propto_{h}+\beta_{1}H_{h,t}+\beta_{2}X_{h,t}+\propto_{t}+\propto_{c}+\;\delta_{c,t}+u_{h,t}\text{,}$$

where ∝ _*h*_ is a dummy variable, which takes the value 1 for household *h* and 0 otherwise. The FE captures the household time‐invariant characteristics and thus avoids a bias that could result from different preferences and human capital endowments that affect health shocks and economic welfare. Compared with the pooled OLS in ((1)), time‐invariant observables Z cancel out. Unobserved time‐invariant household heterogeneity is swept out of the equation. The bias associated with specific household time‐invariant effects that may be correlated with both economic welfare measures, on the left‐hand side, and health shocks, on the right‐hand side, is thus removed. Other variables are as those in ((1)) earlier. The longitudinal sample of the VHLSS does not include households that moved to other communities during 2002–2006; hence, community FEs are swept out of the FE specification. It should be noted that we tested the variance inflation factors of variables included in the models and deemed multicollinearity not to be a problem with all values below the rule of thumb of 5.

While the use of individual FEs will remove time‐invariant heterogeneity and a systematic measurement error, combining FEs with IV may deal with time‐varying heterogeneity and random measurement error. We tested a number of potential instruments for endogenous health variables including district‐level prevalence of health shocks, as well as their interaction with the age and gender of the household head.^11^ As is sometimes the case in the literature (Grimm, 2010), our instruments were weakly correlated with health shocks.^12^

Finally, we investigate if results change across different population groups. Results are presented for the entire sample and by subsample: residential status (urban and rural), female‐headed and male‐headed households, ethnic minority households (all ethnicities other than *Kinh* and *Chinese* 13), and poor and non‐poor households as calculated using the government expenditure‐based poverty line.

## 5 Results

### 5.1 Main results

Descriptive statistics of household expenditures, health shocks, and household characteristics for all households are presented for each wave in Table 1. Appendix S1 shows the descriptive statistics for each subsample in 2008. Expenditures and earned income (expressed in real dongs) move upward over the study period with health expenditures increasing sharply in wave 3. Food expenditures constitute approximately 46% of the total expenditures for the entire sample (Table 1) and 59% for the poor and ethnic minority samples (Appendix S1). The mean number of days in which households were unable to carry out regular activities because of illness or injury is between 9.2 and 12 over the three waves. The mean number of days in bed ranges from 2.4 to 4.1. Table 1 also presents the descriptive statistics for socioeconomic and demographic characteristics of the household. The average size of the household reduces from 4.3 members in period 1 to 4.1 members in period 3.

**Table 1:** Descriptive statistics

	2004		2006		2008	
	Mean	S.E.	Mean	S.E.	Mean	S.E.
Household expenditures and income (in real dong)						
Total household expenditures (non‐health)	17,227.601	(497.228)	22,570.169	(579.914)	30,237.584	(711.302)
Food expenditures	7,862.832	(168.307)	10,479.686	(186.455)	13,401.104	(227.226)
Non‐food expenditures (non‐health)	9,366.769	(353.647)	12,092.484	(442.832)	16,838.479	(538.888)
Education expenditures	947.871	(44.368)	1,305.029	(63.486)	1,577.588	(78.169)
Health expenditures	1,180.039	(141.896)	1,195.734	(82.828)	1,934.864	(207.314)
Income (earned)	19,557.951	(678.139)	27,399.274	(1,051.503)	36,538.623	(1,534.089)
Health shocks of working‐age member(s) of household						
Household with a member unable to carry out regular activities for at least 1 day	0.433	(0.014)	0.438	(0.015)	0.418	(0.014)
Days unable to carry out regular activities because of illness/injury	11.951	(0.916)	11.002	(0.829)	9.169	(0.829)
Household with a member in bed due to illness/injury for at least 1 day	0.188	(0.011)	0.173	(0.010)	0.159	(0.010)
Days in bed due to illness/injury	4.148	(0.575)	2.734	(0.344)	2.395	(0.317)
Household with member hospitalized in the past year	0.153	(0.010)	0.150	(0.010)	0.167	(0.010)
Characteristics of the household						
Household size	4.339	(0.044)	4.266	(0.045)	4.105	(0.045)
Share of household members under age 16 years	0.261	(0.006)	0.234	(0.006)	0.182	(0.005)
Share of household members over age 60 years	0.120	(0.007)	0.130	(0.007)	0.151	(0.007)
Share of male household members	0.492	(0.005)	0.484	(0.005)	0.454	(0.005)
Household lives in a remote commune	0.152	(0.014)	0.130	(0.013)	0.145	(0.014)
Household lives in an urban area	0.220	(0.016)	0.227	(0.016)	0.230	(0.016)
Region of the household						
Red River Delta	0.249	(0.006)	0.249	(0.006)	0.249	(0.006)
Northeast	0.123	(0.003)	0.123	(0.003)	0.123	(0.003)
Northwest	0.024	(0.001)	0.024	(0.001)	0.024	(0.001)
North Central Coast	0.152	(0.005)	0.152	(0.005)	0.152	(0.005)
South Central Coast	0.084	(0.003)	0.084	(0.003)	0.084	(0.003)
Central Highlands	0.036	(0.002)	0.036	(0.002)	0.036	(0.002)
Southeast	0.140	(0.005)	0.140	(0.005)	0.140	(0.005)
Mekong Delta	0.192	(0.005)	0.192	(0.005)	0.192	(0.005)
Month of interview	7.686	(0.074)	7.708	(0.071)	7.399	(0.059)
Change in household head since the previous wave	NA		0.093	(0.008)	0.074	(0.007)
Characteristics of the household head						
Age	49.264	(0.386)	50.395	(0.382)	51.845	(0.361)
Married	0.798	(0.011)	0.803	(0.011)	0.800	(0.011)
Ethnic minority	0.116	(0.010)	0.117	(0.010)	0.116	(0.010)
Male	0.740	(0.012)	0.735	(0.013)	0.726	(0.012)
No formal education	0.283	(0.012)	0.256	(0.012)	0.253	(0.012)
Primary school certificate	0.235	(0.011)	0.241	(0.011)	0.240	(0.011)
Lower secondary school certificate	0.320	(0.012)	0.324	(0.013)	0.322	(0.013)
Upper secondary school certificate	0.124	(0.009)	0.136	(0.010)	0.138	(0.010)
Above secondary education	0.037	(0.006)	0.043	(0.006)	0.044	(0.006)

S.E., standard error; NA, not applicable.

Source: Authors' calculations based on Vietnam Household Living Standards Survey data for years 2004, 2006, and 2008.

Sample size is *N* = 1552 households for each wave. Means are weighted to reflect the complex survey design.

Table 2 reports the OLS and FE specifications of the effect of health shocks on household expenditures and income for the different health problem measures over the entire sample. It gives the coefficient of the health problem measure in the regression of different welfare outcomes. OLS results suggest that health shocks are associated with reduced non‐health household expenditures (total, food, non‐food, and education) and earned income and an increase in health expenditures. When FEs are used, the impact on non‐health household expenditures lessens. The coefficients are small and not statistically significant for the total expenditures and non‐food expenditures. For food expenditures, the coefficient is statistically significant (albeit small) for one health shock measure: days unable to carry out regular activities.

**Table 2:** Effects of health shocks on the log of household expenditures and income

	Pooled OLS			Household FE		
	Days unable to do regular activity	Days in bed	Hospitalization	Days unable to do regular activity	Days in bed	Hospitalization
Total consumption expenditures (non‐health)	−0.027*** (0.006)	−0.022*** (0.007)	−0.035* (0.021)	−0.004 (0.005)	−0.002 (0.006)	0.011 (0.017)
Food expenditures	−0.019*** (0.005)	−0.019*** (0.006)	−0.035** (0.018)	−0.009** (0.004)	−0.004 (0.006)	0.004 (0.016)
Non‐food expenditures (non‐health)	−0.034*** (0.008)	−0.028*** (0.010)	−0.028 (0.029)	−0.001 (0.007)	−0.000(0.009)	0.035 (0.024)
Education expenditures	−0.123*** (0.035)	−0.129*** (0.048)	−0.327** (0.129)	−0.048 (0.031)	−0.107** (0.044)	−0.264** (0.113)
Health expenditures	0.471*** (0.016)	0.584*** (0.022)	1.372*** (0.062)	0.397*** (0.020)	0.484*** (0.027)	1.118*** (0.069)
Total earned income	−0.072*** (0.017)	−0.072*** (0.021)	−0.207*** (0.072)	−0.028** (0.013)	−0.016 (0.016)	−0.027 (0.049)

OLS, ordinary least squares; FE, fixed effects.

Outcomes are transformed into a natural logarithm form. Each coefficient is from a separate regression model. The dependent/independent variable of interest is listed in the row/column heading.

*N* = 1552 households. All regressions include time‐community interaction terms. The OLS regressions include all household and head characteristics shown in Table 1.

The FE regressions include time‐varying regressors only (for more specifics, please see text).

Source: Authors' calculations based on 2004, 2006, and 2008 Vietnam Household Living Standards Survey data.

*** Significance at 1% level;

** Significance at 5% level;

* Significance at 10% level.

For education expenditures, the coefficient is negative, large, and statistically significant for two health measures (days in bed due to sickness and hospitalization). For example, hospitalization of a working‐age member leads to a 26.4% reduction in education expenditures. With all measures, having a health shock leads to a large and significant increase in health expenditures: for instance, a 10% increase in the number of days unable to carry out regular activities because of illness/injury leads to a 4% increase in health expenditures. A significant reduction in earned income is observed for the days unable to carry out regular activities measure, but no significant effect is found for the other two health shock measures.

Table 3 reports the results of the FE specification for the different subsamples and health measures. The results for health expenditures are overall similar for subsamples as for the entire sample, although it is notable that the coefficients of health shocks are smaller for female‐headed households, ethnic minority, and poor households. Several results for subsamples are noteworthy. First, the coefficient of the health shock measure is often consistent in sign across the subsamples but is often not statistically significant in the subsamples, which could be because of the much smaller sample sizes for urban, female‐headed, poor, ethnic minority households. Second, rural households are driving the negative and significant coefficient of the days unable to do regular activity measure for both food expenditures and earned income and of the days in bed and hospitalization coefficients for education expenditures. This is not surprising as the rural sample accounts for 78% of the total sample. Third, female‐headed households,^14^ which comprise 25% of the sample, are particularly affected by days unable to carry out regular activities, which are significantly correlated with lower non‐food and total expenditures. The reduction in education expenditures in response to hospitalization is furthermore the greatest for female‐headed households (significant at the 10% level).

**Table 3:** Fixed effects estimates of the effect of health shocks on the log of household expenditures and income, by household subsample

	All	Rural	Urban	Male	Female	Ethnic	Poor	Non‐poor
Days unable to do regular activity								
Total consumption expenditures (non‐health)	−0.004 (0.005)	−0.008* (0.005)	0.014 (0.012)	0.001 (0.005)	−0.024** (0.010)	−0.000 (0.011)	−0.000 (0.009)	−0.006 (0.005)
Food expenditures	−0.009** (0.004)	−0.011** (0.005)	−0.001 (0.013)	−0.005 (0.005)	−0.020* (0.011)	−0.004(0.011)	−0.006 (0.009)	−0.009* (0.005)
Non‐food expenditures (non‐health)	−0.001 (0.007)	−0.006 (0.007)	0.023 (0.016)	0.006 (0.008)	−0.030** (0.014)	0.006 (0.016)	0.003 (0.015)	−0.004 (0.008)
Education expenditures	−0.048 (0.031)	−0.031 (0.034)	−0.099 (0.076)	−0.042 (0.036)	−0.033 (0.065)	−0.082 (0.073)	−0.123* (0.064)	−0.025 (0.034)
Health expenditures	0.397*** (0.020)	0.422*** (0.022)	0.324*** (0.047)	0.413*** (0.024)	0.369*** (0.043)	0.396*** (0.061)	0.373*** (0.051)	0.404*** (0.022)
Total earned income	−0.028** (0.013)	−0.037*** (0.010)	0.058 (0.053)	−0.022** (0.011)	−0.050 (0.038)	−0.019 (0.015)	−0.003 (0.019)	−0.022 (0.016)
Days in bed								
Total consumption expenditures (non‐health)	−0.002 (0.006)	−0.002 (0.006)	0.001 (0.019)	0.001 (0.006)	−0.013 (0.016)	−0.010 (0.012)	0.001 (0.010)	0.001 (0.007)
Food expenditures	−0.004 (0.006)	−0.005 (0.006)	−0.002 (0.017)	−0.004 (0.006)	−0.005 (0.014)	−0.003 (0.012)	0.000 (0.011)	−0.001 (0.006)
Non‐food expenditures (non‐health)	−0.000 (0.009)	−0.002 (0.009)	0.011 (0.024)	0.004 (0.010)	−0.017 (0.021)	−0.027 (0.021)	0.000 (0.017)	0.003 (0.010)
Education expenditures	−0.109** (0.046)	−0.117** (0.050)	−0.125 (0.128)	−0.120** (0.051)	−0.062 (0.100)	−0.102 (0.110)	−0.083 (0.081)	−0.106** (0.052)
Health expenditures	0.486*** (0.027)	0.510*** (0.029)	0.431*** (0.070)	0.523*** (0.030)	0.387*** (0.057)	0.588*** (0.078)	0.555*** (0.073)	0.483*** (0.030)
Income (earned)	−0.019 (0.017)	−0.020 (0.014)	0.043 (0.072)	−0.027* (0.016)	−0.011 (0.050)	−0.005 (0.020)	0.011 (0.029)	−0.018 (0.020)
Hospitalization								
Total consumption expenditures (non‐health)	0.011 (0.017)	0.000 (0.018)	0.050 (0.048)	0.006 (0.019)	0.007 (0.039)	−0.017 (0.032)	−0.026 (0.028)	0.009 (0.019)
Food expenditures	0.004 (0.016)	−0.004 (0.016)	0.046 (0.055)	−0.008 (0.018)	0.024 (0.043)	−0.009 (0.032)	−0.023 (0.029)	0.001 (0.019)
Non‐food expenditures (non‐health)	0.035 (0.024)	0.025 (0.027)	0.069 (0.054)	0.040 (0.027)	0.004 (0.053)	0.009 (0.057)	0.006 (0.048)	0.033 (0.027)
Education expenditures	−0.264** (0.113)	−0.265** (0.126)	−0.206 (0.324)	−0.141 (0.123)	−0.495* (0.261)	0.041 (0.267)	−0.230 (0.197)	−0.293** (0.125)
Health expenditures	1.118*** (0.069)	1.119*** (0.077)	1.166*** (0.177)	1.165*** (0.082)	1.037*** (0.149)	0.865*** (0.187)	0.861*** (0.205)c	1.143*** (0.074)
Income (earned)	−0.039 (0.052)	−0.035 (0.047)	0.007 (0.219)	−0.044 (0.047)	−0.175 (0.187)	−0.002 (0.052)	0.055 (0.060)	−0.062 (0.060)
*N*	1552	1217	335	1160	392	233	288	1264

Outcomes are transformed into natural logarithms. Each coefficient is from a separate regression model. For each cell, the dependent variable of interest is listed in the row heading.

The coefficient reported is that of the health shock measure (one of three measures given as headers in the first column: ‘Days unable to do regular activity’, ‘Days in bed’, and ‘Hospitalization’). All regressions include time‐community interaction terms.

The fixed effects regressions include time‐varying regressors only (more specifics are in the text).

Source: Authors' calculations based on 2004, 2006 and 2008 VHLSS data.

*** Significance at 1% level;

** Significance at 5% level;

* Significance at 10% level.

Overall, in the entire sample and for most subsamples, households seem to be able to maintain total (non‐health) expenditures despite increased health expenditures. The question then arises as to which mechanisms are used to protect total expenditures. Table 4 presents descriptive statistics on the utilization of selective coping mechanisms. Descriptive statistics are provided for households with or without at least one working‐age member with one or more days unable to carry out regular activities, and the difference between the two groups is tested for statistical significance. Households with health shocks are less likely to receive public pensions or allowances (one‐time sickness or job allowance), but the difference is significant only at the 10% level. There is no statistical difference in the amount of public transfers received for households with and without health shocks. Households with health shocks receive significantly lower private transfers than those without a health shock, particularly international remittances, although a large majority of households receive private transfers irrespective of the health status of household members.

**Table 4:** Descriptive statistics on a possible coping mechanism across household health shock status in 2008

	HH with health shock	HH without health shock	Difference
Public transfers			
% of HH receiving	0.174	0.214	−0.040*
Mean amount received	9550.040	11,874.491	−2324.451
Pension, one time sickness, or job loss allowance			
% of HH receiving	0.082	0.116	−0.035**
Mean amount received	15,063.129	17,817.119	−2753.990
Social welfare allowance			
% of HH receiving	0.107	0.109	−0.002
Mean amount received	4052.221	4317.644	−265.423
Private transfers			
% of HH receiving	0.899	0.895	0.004
Mean amount received	3489.907	5128.432	−1638.525**
Domestic remittances and in‐kind presents received			
% of HH receiving	0.868	0.864	0.004
Mean amount received	2507.952	2998.750	−490.798
International remittances and in‐kind presents received			
% of HH receiving	0.071	0.068	0.003
Mean amount received	13,039.958	29,178.775	−16,138.817**
Charity organizations, associations, and firms			
% of HH receiving	0.054	0.033	0.021*
Mean amount received	755.379	558.418	196.961
Loans			
% of HH who had loans	0.462	0.319	0.143***
Mean amount received	20,575.603	40,625.162	−20,049.559*
Banks or credit organizations			
% of HHs who had loans	0.346	0.239	0.107***
Mean amount received	20,246.442	45,259.389	−25,012.947*
Friends and relatives			
% of HHs who had loans	0.112	0.067	0.045***
Mean amount received	15,213.901	25,659.057	−10,445.156
Other sources^a^			
% of HHs who had loans	0.092	0.045	0.047***
Mean amount received	8701.248	9667.817	−966.569
Asset sales			
% of HHs who sold assets	0.134	0.090	0.043***
Mean value of assets sold	38,572.738	58,623.308	−20,050.570
Productive assets			
% of HHs who sold assets	0.086	0.048	0.037***
Mean value of assets sold	53,108.590	78,767.338	−25,658.748
Gold, silver, and jewelry			
% of HHs who sold assets	0.058	0.042	0.016
Mean value of assets sold	10,668.679	35,521.539	−24,852.860**
Withdrawal of savings			
% of HHs who withdrew savings	0.081	0.072	0.008
Mean amount of savings withdrawn	25,728.301	76,357.774	−50,629.473**
*N*	693	859	1552

HH, household.

Amounts are in real dong and recalled for a 12‐month period. Mean amount received is only among households who received transfer and loan, sold assets, or withdrew savings. Health shock refers to being unable to carry out regular activities because of illness/injury for 1 day or more in the past year.

All estimates are weighted.

a Individual creditors, employment support fund, and others.

*** Significance at 1% level;

** Significance at 5% level;

* Significance of the difference at 10% level.

Households with a health shock are significantly more likely to have loans in total or by specific source (loans from banks, friends/relatives, or other sources) but borrow less in amount. In both cases, formal loans from banks or credit organizations are most common. Households with a health shock are significantly more likely to have sold productive assets: the share of households who sold productive assets is almost twice as high among households with a health problem compared with others (8.6% vs. 4.8%). Finally, households with a health shock are as likely to have made withdrawals from savings in the past year as those without a health shock yet withdrew less.

Overall, these statistics suggest that public and private transfers and withdrawals from savings do not appear to be used by households with a health shock as coping mechanisms, while loans and sale of assets might be. Coping mechanism amounts are further investigated in Table 5 with the FE specification. For public transfers, we find a positive coefficient, but it is not statistically different from zero for two out of the three health shock measures. However, this result changed in the rural and urban subsamples. For private transfers, health measures were largely insignificant. As for loans, a large and significant positive coefficient is found for the hospitalization measure. The coefficient is lower and only significant at 10% level for the other two health measures. For asset sale income, the coefficient is positive and significant for two out of the three health measures (regular activity and hospitalization) for the entire sample and for male‐headed and non‐poor households in particular.^15^ Finally, with respect to savings withdrawal, a significant result was found for rural households for two measures (regular activity and in bed measures) and for poor households for one measure (regular activity measure). As in Table 3, the coefficient of the health shock measure is often not statistically significant in the subsamples, perhaps because of smaller sample sizes, but the sign is mostly consistent across subsamples. One exception is regarding public transfers. A positive coefficient is found for all subsamples^16^ except urban households, where the coefficient is negative for all three health measures.

**Table 5:** Household fixed effects estimates of health shocks on coping mechanisms

	All	Rural	Urban	Male	Female	Ethnic	Poor	Non‐poor
Days unable to do regular activity								
Public transfers	0.019 (0.025)	0.058** (0.026)	−0.191** (0.080)	0.016 (0.031)	0.033 (0.048)	0.056 (0.097)	0.043 (0.071)	0.006 (0.027)
Private transfers	0.039 (0.036)	0.069* (0.040)	−0.055 (0.098)	0.068* (0.040)	−0.043 (0.087)	0.117 (0.111)	0.013 (0.097)	0.033 (0.036)
Loans	0.098* (0.056)	0.139** (0.064)	−0.037 (0.118)	0.082 (0.068)	0.161 (0.115)	0.214 (0.133)	0.196 (0.130)	0.114* (0.066)
Asset sale income	0.124** (0.054)	0.112* (0.064)	0.179* (0.099)	0.148** (0.062)	0.068 (0.118)	−0.102 (0.117)	−0.069 (0.122)	0.137*** (0.053)
Saving withdrawal	0.033 (0.038)	0.081** (0.041)	−0.147 (0.094)	0.064 (0.047)	−0.097 (0.074)	0.118* (0.066)	0.121** (0.059)	0.014 (0.038)
Days in bed								
Public transfers	0.024 (0.039)	0.063* (0.037)	−0.164 (0.136)	0.023 (0.045)	0.048 (0.078)	0.068 (0.076)	0.038 (0.070)	0.012 (0.044)
Private transfers	0.053 (0.043)	0.068 (0.048)	−0.042 (0.104)	0.048 (0.049)	0.055 (0.093)	0.020 (0.120)	0.009 (0.128)	0.067 (0.047)
Loans	0.147* (0.077)	0.131 (0.086)	0.298* (0.177)	0.105 (0.088)	0.273 (0.171)	−0.301 (0.200)	−0.075 (0.200)	0.204** (0.083)
Asset sales	0.105 (0.067)	0.133* (0.079)	0.017 (0.115)	0.121* (0.072)	0.079 (0.164)	−0.113 (0.128)	0.010 (0.127)	0.130* (0.077)
Savings withdrawal	0.034 (0.044)	0.096** (0.046)	−0.267** (0.130)	0.021 (0.056)	0.133 (0.083)	0.088 (0.071)	0.086 (0.069)	0.025 (0.050)
Hospitalization								
Public transfers	0.196** (0.084)	0.230** (0.091)	−0.139 (0.234)	0.183* (0.101)	0.321* (0.184)	0.226 (0.222)	0.293 (0.223)	0.173** (0.087)
Private transfers	−0.008 (0.115)	−0.022 (0.128)	0.219 (0.320)	0.087 (0.137)	−0.331 (0.228)	−0.465 (0.284)	−0.600** (0.292)	0.114 (0.126)
Loans	0.476** (0.216)	0.507** (0.241)	0.378 (0.535)	0.354 (0.247)	0.857* (0.504)	0.075 (0.460)	−0.120 (0.592)	0.584** (0.228)
Asset sales	0.331** (0.156)	0.407** (0.183)	−0.114 (0.299)	0.611*** (0.188)	−0.542** (0.269)	0.353 (0.422)	0.004 (0.403)	0.433** (0.168)
Savings withdrawal	0.176 (0.129)	0.137 (0.140)	0.227 (0.372)	0.198 (0.157)	0.077 (0.224)	−0.003 (0.161)	0.084 (0.283)	0.197 (0.149)
*N*	1552	1217	335	1160	392	233	288	1264

Outcomes are transformed into natural logarithms. Each coefficient is from a separate regression model. For each cell, the dependent variable of interest is listed in the row heading. The coefficient reported is that of the health shock measure (one of three measures given as headers in the first column: ‘Days unable to do regular activity’, ‘Days in bed’, and ‘Hospitalization’).All regressions include time‐community interaction terms.

Source: Authors' calculations based on 2004, 2006 and 2008 VHLSS data.

*** Significance at 1% level;

** Significance at 5% level;

* Significance at 10% level.

### 5.2 Robustness checks

Our results are robust to attrition. Attrition can be a problem if observable or unobservable factors that result in attrition are correlated with the error term in the specification of interest (Fitzgerald *et al.*, 1998). Attrition in the VHLSS panel over the three waves is 22%: 11% between waves 1 and 2 and 12% between waves 2 and 3. None of the health measures under study were significantly correlated with the probability of attrition.^17^ Results on the determinants of sample attrition are presented in Appendix S2. We also did the analysis using only two waves of data (2004–2006 and 2006–2008) and thus with less attrition and a larger sample size. The main results on consumption smoothing and coping mechanisms were robust. However, the negative impact of health shocks on non‐health expenditures for female‐headed households was not significant for the 2004–2006 or 2006–2008 samples.

The finding on the reduction in education expenditures could be due to health shocks experienced by children in the household and perhaps correlated with those experienced by working‐age adults. As a robustness check, we added as a control a variable in equation (2) to indicate that at least one child in the household had a health shock. The coefficient of the health shock status for working‐age members of the household remained negative and statistically significant, indicating that the reduction in education expenditures is not driven by health shocks among children.

Finally, although this paper is not aimed at analyzing the effects of health insurance programs and model ((2)) does not address the endogeneity of health insurance, we repeated the analysis by adding health insurance status as a time‐varying control variable. Results are presented in Appendices S3 and S4 and are overall very similar to those in Tables 3 and 5, respectively. In particular, results in Appendix S3 on the log of household expenditures and income are very similar to those in Table 2, except for the hospitalization measure where the coefficient is higher in absolute value in the education expenditures specification.

## 6 Discussion

This paper offers several important findings with implications for future research and policy. Using the three measures of health shocks with three waves of national living standards survey data in Vietnam, we find that households face a significant rise in health expenditures when they experience a health shock. Results on earned income are mixed: earned income is reduced because of a limitation in regular activity measure but not for the other two health shock measures. These results on health expenditures and income are consistent with those in an earlier study in Vietnam (Wagstaff, 2007a). However, our results differ from this earlier study in one important respect. Using 1993–1998 data for Vietnam, Wagstaff (2007a) finds that households are unable to insure consumption against several health measures, including a hospitalization measure as used in this paper. In contrast, we find that households are able to maintain total non‐health expenditures, except for female‐headed and rural households. This suggests that the impact of health shocks on non‐health expenditures in Vietnam is lesser than it was in the 1990s.

The contribution of our paper is that we explore possible explanations through the responsiveness of a wide range of coping mechanisms to the health problems of working‐age members. The primary means through which households insure consumption is increasing loans and the sale of assets, followed by a reduction in education expenditures. The results on loans and the sale of assets are consistent with other studies (Nguyen *et al*., 2012), while the reduction in education expenditures is, to our knowledge, a new finding in this literature.

Overall, our results support the case made by Chetty and Looney (2006) that smooth consumption is not an adequate indicator for the need for social insurance as households may adopt coping strategies that make them more vulnerable. The bulk of loans in Vietnam are sourced from banks and formal credit organizations. Interest‐bearing loans pose a threat of poverty and reduce the ability of households to cope with future shocks. The majority of assets sold were productive assets, comprising the future ability of households to generate a livelihood and manage risk. Cutting education expenditures may have long‐term and intergenerational effects on human capital formation for the household, as the welfare returns to investment in human capital in LMICs are high (Patrinos and Psacharopoulos, 2010). Our results overall also support the finding that health shocks contribute to households descending into poverty (Krishna, 2010).

In addition, this result is consistent with the broader literature on the two‐way causal links between health and education and the intergenerational transmission of low human capital and educational gradients in health. Our result points to one possible intergenerational channel, whereby health may influence education: the health shocks of working‐age adults. It is also consistent with a recent study of basic health insurance in rural China, where health insurance was found to have an effect on school enrollment for children (Chen and Jin, 2010).

Public and private transfers, by and large, were found to be unresponsive to health shocks. Limited reliability on public transfers seems to be consistent with the result by Van‐den‐Berg and Nguyen (2011) that public transfers' impact on poverty and inequality in the early 2000s was low, because of low coverage of the poor and relatively low amounts transferred to the poor. In our sample, approximately 10% of households were classified by local authorities as poor and therefore entitled to monthly income support. This compares with our calculated rate of 19% using the government expenditure‐based poverty line.

The expansion of social health insurance has been a major policy undertaking in Vietnam. To date, approximately 60% of the population is covered by insurance. While evaluating the impact of health insurance was beyond the scope of this paper, our results remained little changed when controlling for insurance. One explanation is that over half of rural households, who made up the bulk of the sample, had working‐age members that were uninsured (51%).^18^ Following Wagstaff (2007a), we also extended our analysis to assess the differences in health spending by health insurance status. Like this earlier paper, we find that the effect of a health shock of a working member on medical spending is larger for the uninsured than the insured across all samples.

These results suggest that more has to be carried out to expand and deepen health insurance coverage, particularly in rural areas. Rural households are most unable to shield earned income and consumption expenditures in the face of health shocks and are most prone to draw upon a wider range of coping mechanisms, including public transfers and welfare‐detrimental strategies such as reduction in education expenditures and the uptake of loans, followed by the sale of assets and, to a lesser extent, withdrawal of saving. Health shocks tend to trigger asset sales and loans more than public transfers in rural areas. We did find that public transfers were responsive to health shocks in rural areas, which may be explained by the expansion of the unconditional cash transfer program for the poor and social beneficiary groups who live in higher proportion in rural areas (Decree 67, 2007).

The expansion of income support to the working near poor in rural areas is put forward as a consideration for policymakers. Income support can provide an important buffer to non‐health consumption expenditures while also contributing to ongoing private health‐related expenditures, such as those relating to medication and rehabilitation, which are not covered by formal health insurance (Hoang *et al*., 2015).

For poor households, we find that they are able to shield their welfare from health shock without undertaking loans or selling productive assets. This result is contrary to the study in Dai Dong province, Vietnam (Nguyen *et al*., 2012). Our result holds true for ethnic minority households and may be attributed to targeted social health insurance and public programs for these groups. The health insurance program targeted at the poor (Health Care Fund for the Poor) has been subject to a number of evaluations, which suggest that the program led to reduced out of pocket medical expenditures (Wagstaff, 2007b, 2010; Axelson *et al*., 2009). The result may also reflect low levels of asset ownership, which may serve as collateral for loans. Indeed, non‐poor households were more likely to sell assets in the event of a health shock. By one health measure (regular activity), poor households withdraw savings and reduced education expenditures (significant at the 10% level only), which suggests further that these households had fewer coping mechanisms available to them.

For women‐headed households, this study has a new and worrisome finding. We find a significant drop in the total non‐health expenditures following the health shock of a working‐age member. We furthermore find the largest uptake of loans and a reduction in education expenditures following the hospitalization of a working member. Female‐headed households are smaller in size than their male counterparts (3.8 versus 4.5 members, respectively), which suggests that they are less able to substitute labor within the household. Furthermore, our results on private transfers suggest that they have lower levels of social capital. Overall, the findings paint a precarious picture for the welfare of these households following a health shock, in both the short term and the longer term. They suggest further that female‐headed households be included as among the legislated social beneficiary groups in Vietnam.

Our results are consistent with a study of 15 LMICs that finds, in most countries, similar non‐health expenditures across disability status and yet, households with disabilities are more likely to experience high health expenditures and multidimensional poverty (Mitra *et al*., 2013). In other Asian countries, health shocks have also been shown to significantly increase health expenditures. However, unlike the results in this paper, effects were larger on income than on health expenditures (Gertler and Gruber, 2002). Our result on the ability to insure non‐health expenditures through the use of assorted household coping mechanisms is consistent with the findings from Indonesia (Genoni, 2012; Sparrow *et al*., 2013) and Bangladesh (Islam and Maitra, 2012). Heterogeneity in the ability to insure consumption across population groups is consistent with the results of Sparrow *et al*. (2013) for the poor in Indonesia.

The experience of Vietnam suggests that it is critical to extend social health insurance to the rural near‐poor population in order to mitigate the welfare risks associated with idiosyncratic health shocks. In spite of significant state subsidies, the uptake of voluntary insurance among the working near poor has been slow. Out‐of‐pocket payments furthermore are significant for the insured in Vietnam, and the country experiences among the highest rates of catastrophic health spending in the world with out‐of‐pocket payments representing the dominant source of healthcare financing (Xu *et al*., 2003). In the pursuit of universal health coverage, it is thus equally important to ensure that sufficient financial protection is provided from the costs of care. Third, the ability of households to cope in the event of a health shock to a working‐age member is not homogenous across the population. There exists some evidence in the case of Vietnam that targeted social health insurance programs may provide some relief. As a complement to social health insurance, there exists a potentially important role for public transfers to act as a buffer for consumption and private healthcare expenditures. Access to low‐interest loans and microcredit may furthermore help alleviate vulnerability to health problems as found in other LMICs settings (Islam and Maitra, 2012).

This paper points toward possible avenues for further research. We find that results vary to some extent across different health shock measures. For the entire sample, several results hold for all three measures: the absence of a significant change in the total non‐health expenditures and non‐food expenditures, income from private transfers and savings withdrawals, and the significant increase in health expenditures and income from loans. The other main results hold for two measures: the reduction in education expenditures, the increase in income from asset sales and the lack of a significant effect on income from public transfers. This confirms the importance of including multiple health measures in surveys. Unfortunately, many surveys continue to use health measures that are problematic for the study of the socioeconomic determinants or consequences of health shocks, such as diagnosis data. Furthermore, data on mental health and on a broad range of functionings, as recently advised by Washington Group on Disability Statistics, are very rare (Madans *et al*., 2010).

In addition, as noted earlier, the coping mechanisms used by households to mitigate the impact of health shocks on household welfare may be of concern. Households that cope with health shocks through savings, borrowing, or the sale of assets may not experience significant economic losses in the short term compared with those without access to these mechanisms. However, the medium‐ and long‐term impacts on welfare and human capital may be significant because of reduced investment capital, high‐interest repayments, and loss of productive capacity. Many studies are limited by short‐term or retrospective survey data. For example, Gertler and Gruber (2002) and Wagstaff (2007a) use two waves of panel data; Wagstaff and Lindelow (2014) and Heltberg and Lund (2009) use retrospective modules that are subject to recall bias (Ravallion, 2014). Further research is needed on the medium‐term and long‐term consequences of health shocks.

Of course, the analysis earlier is not without limitations. A major limitation of the analysis in this paper is that no IV could be used; hence, results may be biased because of random measurement error and unobserved time‐varying heterogeneity. Another limitation lies in the limited set of health measures that are available in the data under use. We also returned some slightly unusual results, such as the negative coefficient on public transfers in the urban subsample, which likely reflects estimation difficulties associated with small sample size. The relatively small size of the panel sample over three waves of data presents a limitation for the analysis of smaller subsamples.

## 7 Conclusion

According to our results, as Vietnam was experiencing increasing incomes that led to its middle‐income country status in 2010 and expanding its social protection system, health shocks in the working‐age population had far‐reaching economic consequences for Vietnamese households over the period 2004–2008. While households managed to smooth non‐health consumption, they had to cope with increased health expenditures with no significant reliance on public/private transfers, especially in rural areas. Households adopted a number of vulnerability‐enhancing coping mechanisms including cutting education expenditures, taking on loans, and selling assets. Public transfers, including cash transfers and pensions, should be further expanded and deepened in coverage as few households use public transfers as coping mechanisms. Our results also strongly support efforts to expand access to social health insurance and reduce the costs of education to households.

## Conflict of Interest

The authors have no conflict of interest.

## Ethical Statement

No ethical approval was needed for the conduct of this study.

## Supporting information



Supporting info item

## Footnotes

1 For this reason, several recent papers have used terms other than health shocks, such as ‘ill health events’ (Sparrow *et al*., 2013).

2 Some studies adopt a hybrid specification of the lagged and a first difference specification that partially addresses a systematic measurement error by taking the first difference of health measures and welfare outcomes. For example, Gertler and Gruber (2002) regress the changes in consumption outcomes on the changes in health and a vector of control characteristics. These control characteristics, though, are observed at baseline, and so, this is not a full first difference specification that would address a systematic measurement error and time‐invariant heterogeneity.

3 It should be noted that both Wagstaff (2007a) and Genoni (2012) find significant effects for income but not consumption.

4 Wagstaff (2007a) furthermore finds that household earned income is negatively affected by the death of a working‐age member but not a drop in BMI or an inpatient stay of more than a week.

5 For example, Le and Booth (2014) find that between 1993 and 2006, the mean per capita expenditure of urban households is consistently double that of rural households with the gap increasing from poorer to richer groups. Kang and Imai (2012) find that while over the period 2002–2006, poverty reduction for ethnic minorities exceeded the majority, over 50% of the ethnic minorities remained poor, and the gap in living standards has been widening.

6 Refer to the works of Ekman *et al*. (2008) and Lieberman and Wagstaff (2009) for a detailed review on the development of social health insurance in Vietnam.

7 Medical expenditures include those associated with inpatient and outpatient contacts, non‐prescription medication, and medical equipment.

8 The classification of remote commute changes over time.

9 Community information is available at different levels of geographic aggregation: commune, district, province, and region levels. Cell sizes were sometimes too small to use commune or district fixed effects. Results presented in this paper are for province fixed effects. There are 64 provinces in Vietnam.

10 We conducted panel bootstrap Hausman tests that strongly rejected the null hypothesis that random effects provide consistent estimates (Wooldridge, 2002). For example, for the robust Hausman test of the logged total consumption expenditures (non‐health), outcome on a subset of key time‐varying regressors is as follows: number of days unable to do regular activity of working‐age household members; household size; proportion of household members under the age of 16 years; proportion of household members over the age of 60 years; proportion of male household members; and year dummies.

11 Information on prices of medical services or food has been used in the literature (e.g., Genoni, 2012; Shultz and Tansel, 1997) but is not available in the Vietnam Household Living Standards Survey.

12 For example, the first stage F‐statistic for the instrument natural log of average number of days unable to perform regular activities of a working‐age member in the district was 1.23. Instruments for public transfers were similarly weak. When first stage F‐statistics are small, two‐stage least square estimates and CIs are unreliable (Staiger and Stock, 1997).

13 There exist 54 ethnic groups in Vietnam. The *Kinh* Vietnamese account for 86% of the national population and display similar socioeconomic characteristics as the Chinese (Hoa). Thus, the two ethnic groups are often classified together as the ethnic majority (Kang and Imai, 2012).

14 Female‐headed households are households with a woman reported as head. These female heads include widows (46%), spouses of migrant workers (41%), divorced or separated (7%), and never‐married women (5%).

15 It is unclear why the coefficient is negative for women, but this is the case only for the hospitalization measure.

16 For rural households, the coefficient is positive and significant, albeit at 10% level only for the days in bed measure.

17 We also checked for each wave that the weighted means for the panel sample, the non‐panel sample, and the cross‐sectional sample (which is nationally representative) are overall similar for the dependent and independent variables.

18 This compares with only 41% of households in urban areas who had no insured working‐age members.
